# Novel *CYP27B1* Gene Mutations in Patients with Vitamin D-Dependent Rickets Type 1A

**DOI:** 10.1371/journal.pone.0131376

**Published:** 2015-07-01

**Authors:** Korcan Demir, Walaa E. Kattan, Minjing Zou, Erdem Durmaz, Huda BinEssa, Özlem Nalbantoğlu, Roua A. Al-Rijjal, Brian Meyer, Behzat Özkan, Yufei Shi

**Affiliations:** 1 Division of Pediatric Endocrinology, Dr. Behçet Uz Children’s Hospital, İzmir, Turkey; 2 College of Science and General Studies, Alfaisal University, Riyadh, Saudi Arabia; 3 Department of Genetics, King Faisal Specialist Hospital & Research Centre, Riyadh, Saudi Arabia; 4 Department of Pediatric Endocrinology, Sifa University, Bornova Health Application and Research Center, İzmir, Turkey; Odense University Hospital, DENMARK

## Abstract

The *CYP27B1* gene encodes 25-hydroxyvitamin D-1α-hydroxylase. Mutations of this gene cause vitamin D-dependent rickets type 1A (VDDR-IA, OMIM 264700), which is a rare autosomal recessive disorder. To investigate *CYP27B1* mutations, we studied 8 patients from 7 unrelated families. All coding exons and intron-exon boundaries of *CYP27B1* gene were amplified by PCR from peripheral leukocyte DNA and subsequently sequenced. Homozygous mutations in the *CYP27B1* gene were found in all the patients and heterozygous mutations were present in their normal parents. One novel single nucleotide variation (SNV, c.1215 T>C, p.R379R in the last nucleotide of exon 7) and three novel mutations were identified:, a splice donor site mutation (c.1215+2T>A) in intron 7, a 16-bp deletion in exon 6 (c.1022-1037del16), and a 2-bp deletion in exon 5 (c.934_935delAC). Both c.1215 T>C and c.1215+2T>A were present together in homozygous form in two unrelated patients, and caused exon 7 skipping. However, c.1215 T>C alone has no effect on pre-mRNA splicing. The skipping of exon 7 resulted in a shift of downstream reading frame and a premature stop codon 57 amino acids from L380 (p.L380Afs*57). The intra-exon deletions of c.1022-1037del16 and c.934_935delAC also resulted in a frameshift and the creation of premature stop codons at p.T341Rfs*5, and p.T312Rfs*19, respectively, leading to the functional inactivation of the *CYP27B1* gene. Clinically, all the patients required continued calcitriol treatment and the clinical presentations were consistent with the complete loss of vitamin D1α-hydroxylase activity. In conclusion, three novel mutations have been identified. All of them caused frameshift and truncated proteins. The silent c.1215 T>C SNV has no effect on pre-mRNA splicing and it is likely a novel SNP. The current study further expands the *CYP27B1* mutation spectrum.

## Introduction

Vitamin D (calciferols) is a group of biologically inactive, fat-soluble pro-hormones. Two forms of vitamin D exist: ergocalciferol (vitamin D_2_) and cholecalciferol (vitamin D_3_). Vitamin D_2_ is derived from plants and vitamin D_3_ is produced by animal tissues or from the conversion of 7-dehydrocholesterol in the human skin by the solar Ultraviolet B (UVB) radiation at the wavelength of 280–315 nm [1]. Both forms of vitamin D need two-step hydroxylation to become biologically active. The first step occurs in the liver where vitamin D is hydroxylated to 25-hydroxyvitamin D (25OHD) by several hepatic enzymes having 25-hydroxylase activity, such as microsomal *CYP2R1*[2] and *CYP3A4* [3], and mitochondrial *CYP27A1*[4]. The CYP2R1 is, however, the major enzyme for hydroxylation of vitamin D to 25-hydroxyvitamin D [5]. The 25-hydroxylation in the liver is very efficient, converting most vitamin D to 25OHD on a single pass through the liver. The serum half-lives of vitamin D, 25OHD, and 1, 25-(OH)_2_D is about 24 hours [6], 2–3 weeks [7] and 4 hours [8, 9], respectively. The long half-life of 25OHD is due to high affinity of vitamin D binding protein to 25OHD [10]. As a result, serum level of 25OHD is about 1000-fold higher than that of 1, 25-(OH)_2_D. The second step of hydroxylation occurs mainly in the kidney, where 25(OH)D is hydroxylated by the mitochondrial 25-hydroxyvitamin D-1α-hydroxylase to the biologically active hormone 1, 25-(OH)_2_D, which binds to and activates vitamin D receptor [1]. The mitochondrial 25-hydroxyvitamin D-1α-hydroxylase activity is also found in extra-renal tissues such as skin, gastrointestinal tract, thyroid, testes, brain, osteoblasts, macrophages and placenta [11, 12]. The biologically active 1, 25-(OH)_2_D plays a central role in calcium homeostasis and bone metabolism, and also has a significant influence on cell proliferation and differentiation of a variety of tissues [1, 13, 14]. The renal synthesis of 1,25-(OH)_2_D from its precursor, 25(OH)D is a rate-limiting step and is tightly regulated by serum1,25-(OH)_2_D, parathyroid hormone (PTH), FGF23, calcium, and phosphate, with renal 1α-hydroxylase being stimulated by PTH, hypophosphatemia, or hypocalcaemia, and inhibited by FGF23 [1, 14, 15].

Vitamin D-dependent rickets type 1A (VDDR-IA) is a rare autosomal recessive disorder caused by mutations in the *CYP27B1* gene, which encodes 25-hydroxyvitamin D-1α-hydroxylase [16]. It is characterized clinically by hypotonia, growth retardation, muscle weakness, hypocalcemic seizures in early infancy, and radiographic features of rickets. Typical laboratory findings include hypocalcemia, elevated serum PTH, and low-normal, low or undetectable serum 1, 25(OH)_2_D despite normal or increased serum 25OHD [14, 17–19]. More than 100 patients with 64 different mutations have been reported from different ethnic groups [16, 19–26].Certain mutations are more frequent in certain ethnic groups [19, 24, 25].

In the present study, we report 8 patients with vitamin D 1α-hydroxylase deficiency from 7 unrelated Turkish families. Both novel and previously reported mutations in the *CYP27B1* gene have been found.

## Subjects and Methods

### Patients

Eight patients with rickets and their parents were recruited for the study. Vitamin D dependent rickets type 1A was considered clinically when rickets was associated with hypo- or normocalcemia, hypophosphatemia, hyperphosphatasemia, markedly elevated serum PTH, normal or high vitamin D levels, and low or inappropriately normal calcitriol levels. Five patients were from consanguineous and 3 from non-consanguineous Turkish families. All of the parents were asymptomatic and free of rickets. Wrist radiographs of all of the patients demonstrated delayed bone age and coarse trabeculation, growth plate widening and cupping and fraying of the metaphyseal regions of ulna and radius. None of the patients developed nephrocalcinosis during follow-up. The clinical and laboratory data for these patients are summarized in Table 1. The study and consent form were approved by the Research Ethics Committee of King Faisal Specialist Hospital and Research Centre. Written consent was obtained from the guardians of the patients before enrollment.

**Table 1:** Clinical, laboratory, and genetic findings of 7 families with VDDR-I.

Family	Subjects	Clinical features	Time point	Age	Height SDS	Ca mg/dL	P mg/dL	ALP IU/L	25OHD ng/ml	1,25(OH)_2_ D pg/mL	PTH ng/L	Mutation
I*	I-1Father	normal										heterozygousc.1022-1037del16
	I-2Mother	normal										heterozygousc.1022-1037del16
	I-3Son	Inability to walk	At diagnosis	16 months	-2.5	8.5	3.4	1802	40.44	3.2	703.8	homozygous c.1022-1037del16
			Most recent	25 months	-2.87	9.5	3.4	1131	ND	ND	195.3	homozygous c.1022-1037del16
II**	II-1Father	normal										heterozygousc.1022-1037del16
	II-2 Mother	normal										heterozygousc.1022-1037del16
	II-3Son	Failure to thrive and inability to walk	At diagnosis	17 months	-2.85	8.9	1.94	1523	189	9.1	560	homozygous c.1022-1037del16
			Most recent	25 months	-2.87	9.1	3.2	638	52.8	ND	136.6	homozygous c.1022-1037del16
III	III-1 Father	normal										heterozygousc.1215+2T>A
	III-2 Mother	normal										heterozygousc.1215+2T>A
	III-3Son	Inability to walk	At diagnosis	21 months	-4.13	6.5	2.9	1622	125	25	319	homozygousc.1215+2T>A
			Most recent	8 years	-2.45	9.4	4.6	226	23.1	ND	28	homozygousc.1215+2T>A
IV*	IV-1 Father	normal										heterozygousc.195+2T>G
	IV-2 Mother	normal										heterozygousc.195+2T>G
	IV-3 Son	Failure to thrive and inability to walk	At diagnosis	12 months	-2.54	8.9	1.8	2190	44	4.5	938	homozygousc.195+2T>G
			Most recent	16 months	-3.29	8.7	2.2	1879	ND	ND	998	homozygousc.195+2T>G
	IV-4 Daughter	Failure to thrive and inability to walk	At diagnosis	26 months	-5.22	7.1	2.7	1850	35	<2.1	466	homozygous c.195+2T>G
			Most recent	8 years	-5.28	9.2	5.2	343	40.7	ND	138	homozygous c.195+2T>G
V	V-1 Father	normal										heterozygousc.195+2T>G
	V-2 Mother	normal										heterozygousc.195+2T>G
	V-3 Daughter	Failure to thrive and fractures	At diagnosis	21 months	-3.96	8.6	2.5	1825	238	14	728	homozygous c.195+2T>G
			Most recent	31 months	-3.72	10.2	4.1	432	41.3	ND	37.7	homozygous c.195+2T>G
VI*	VI-1 Father	normal										heterozygousc.1215+2T>A
	VI-2 Mother	normal										heterozygous c.1215+2T>A
	VI-3 Daughter	Failure to thrive, fractures, and blue sclera	At diagnosis	13 months	-4.33	4.2	3.5	684	40	ND	284	homozygous c.1215+2T>A
			Most recent	12 years	-4.5	9.0	5	232	31.7	ND	217	homozygous c.1215+2T>A
VII*	VII-1 Father	normal										heterozygous c.934_935delAC
	VII-2 Mother	normal				9.4	4	86	12.2	50	50	heterozygous c.934_935delAC
	VII-3 Daughter	Hypocalcemic convulsion	At diagnosis	13 months	ND	6.5	3.9	1100	54	13	555	homozygousc.934_935delAC
			Most recent	25 months	-1.3	9.6	4	350	ND	ND	40	homozygousc.934_935delAC
Normal range						8.8–10.6	3.7–6.8	82–380	20–100	17–53	15–65	

ND: not done; SDS: standard deviation score or Z-score

SI unit conversions: to convert the values for 25OHD to nmol/L, multiply by 2.5; to convert the values for 1,25(OH)_2D_ to pmol/L, multiply by 2.4; to convert the value for calcium to mmol/L, divide by 4; to convert the values for phosphate to mmol/L, divide by 3.1.

*: consanguineous families.

**: non-consanguineous family, but parents from the same village. Family III and VI are not related.

#### Patient I-3

The 16 month-old male patient was admitted due to inability to walk. Dental eruption and independently sitting occurred, but slightly delayed. Physical examination was normal other than short stature, genu varum, and widening of wrists. He was able to walk three months after initiation of calcitriol (60 ng/kg/day).

#### Patient II-3

The 17 month-old male patient presented with failure to thrive and delay in developmental milestones. No clinical improvement had been observed with a single-day high-dose vitamin D treatment. Unilateral genu varum and widening of wrists were noted on physical examination. After five months of treatment with calcitriol (60 ng/kg/day), calcitriol dose was increased to 85 ng/kg/day, which resulted in fast amelioration of clinical and biochemical abnormalities.

#### Patient III-3

The 21 month-old male patient presented with growth retardation and inability to walk. Dental eruption was also delayed. Physical examination revealed frontal bossing, rachitic rosary and widening of wrists. Biochemical values returned to normal after six months of treatment using 100 ng/kg/day calcitriol. At his most recent visit at the age of 7-¾ years, his height was 113.5 cm (-2.45 standard deviation score, SDS or Z-score) and physical examination, biochemical values, and wrist radiograph were normal while on 50 ng/kg/day calcitriol and 75 mg/kg/day calcium lactate. His parents are relatively short and target height is 166 ± 5 cm (-1.44 SDS).

#### Patient IV-3

The patient is younger brother of Patient IV-4. He presented at the age of 1 year with growth failure and inability to sit or walk. Rachitic rosary and widening of wrists were evident. He was treated with alfacalcidol (130 ng/kg/day) and calcium lactate (75 mg/kg/day), but half of the recommended dose was reported to be used. Though, he is now able to sit.

#### Patient IV-4

The patient presented with growth failure and delay in developmental milestones at the age of 26 months. She had been given several doses of vitamin D without clinical benefit. Physical examination showed hypotonia, frontal bossing, rachitic rosary, widening of wrists, and an anterior fontanelle 1x1 cm in size. She was treated with calcitriol 140 ng/kg/day. Compliance to the treatment was poor, resulting in lack of clinical follow-up between 3 and 5 years of age, and lack of improvement in growth. At her most recent visit at the age of 8 years, she was using calcitriol (30 ng/kg/day) and calcium lactate (50 mg/kg/day), was remarkably short (99.9 cm, -5.28 SDS), and had genu valgum, scoliosis and a mildly elevated PTH level (138 ng/L).

#### Patient V-3

This patient is the most severely affected one among our patients. Delay in developmental milestones was first noted at 6 months of age. At presentation, she was on calcitriol treatment, which was used intermittently. Physical examination showed caput quadratum, large anterior fontanelle, rhizomelia, rachitic rosary and widening of wrists. Radiological evaluation demonstrated healing fractures and bowing of femura and humeri. Alfacalcidol (120 ng/kg/day) and calcium lactate (100 mg/kg/day) were started and her biochemical and clinical improvement including respiratory function were observed. The patient also had respiratory problems that were attributed to gastroesophagial reflux.

#### Patient VI-3

The patient was diagnosed to have rickets at the age of six months and repeated doses of high-dose vitamin D treatment resulted in transient improvement in biochemical values. At 13 months of age, she presented to our department with short stature and hypotonia. Physical examination disclosed blue sclera, rachitic rosary, and widening of wrists. Radiological evaluation demonstrated a distal radius fracture in addition to rickets. Calcitriol treatment was started with a clinical diagnosis of 1α-hydroxylase deficiency. Subsequently, she was lost to follow-up for 2 years. She came back with several fractures due to poor compliance to treatment. Normalization of biochemical values and PTH level could be achieved, but only twice. At her most recent visit at the age of 11-¾ years, she was taking alfacalcidol (50 ng/kg/day) and calcium lactate (50 mg/kg/g), was remarkably short 115 cm (-4.50 SDS), and had genu valgum, mild scoliosis, and high PTH level (215.7 ng/L). Parathyroid scintigraphy regarding possible tertiary hyperparathyroidism was negative.

#### Patient VII-3

The 13-month old patient presented with hypocalcemic seizures. Her serum calcium and 1, 25 (OH)_2_ vitamin D were 6.5 mg/dL and 13 pg/mL, respectively. She was started on calcium (50 mg/kg/day) and oral calcitriol (0.5 μg/day). Her calcium level returned to normal (8.9 mg/dL) one month later and she never had seizure again.

### Genomic DNA isolation

Genomic DNA from peripheral blood leukocytes was isolated using the Gentra Blood Kit (Qiagen Corp, CA).

### DNA amplification and sequencing

All the 9 exons and intron-exon boundaries of the *CYP27B1* gene (NM_000785) were amplified by PCR from 100 ng of genomic DNA as described previously [26]. The resulting PCR products were directly sequenced by bidirectional Sanger sequencing using an automated ABI PRISM 3700 sequencer (Foster City, CA). The primer sequences are available upon request.

### RNA extraction and RT-PCR

Total RNA was extracted from peripheral blood leukocytes by quanidine thiocyanate-phenol-chloroform method [27]. Two μg of total RNA were reverse-transcribed into cDNA using Promega reverse transcription system (Promega, Madison, WI). To improve specificity, nested RT-PCR was used to amplify *CYP27B1* transcripts covering exons 6–8, first using the following two primers: 5’-gtgtccaacacgctctcttg-3’ (forward primer located in exon 6), and 5’-atacagctgcgcttgccaaagc-3’ (reverse primer located in exon 8). The resulting PCR products were re-amplified by PCR using internal forward primer (5’-gcactccactcagagatcacag -3’ in exon 6) and the reverse primer. The PCR conditions were 95°C for 5 min followed by 35 cycles of amplification (95°C for 40s, 54°C for 40s, and 72°C for 40s) with final extension of 5 min. The resulting PCR products were analyzed by gel electrophoresis and were subsequently sequenced.

### Mini-gene construction

To determine the effect of c.1215 T>C on pre-mRNA splicing, we created a mini-gene by PCR amplification of a 940-bp wild-type genomic DNA fragment containing exons 6–8 and introns 6–7 using the following primers: 5’-gtgtccaacacgctctcttgggc-3’ and 5’-ctgggccaaagccatttgcaat with 30 cycles of PCR amplification: 95°C for 1 min, 52°C for 1 min, and 72°C for 1 min. The resulting PCR products were subcloned into pcDNA3.1 vector (*CYP27B1* ^WT^). The c.1215 T>C was created by site-directed mutatgenesis (*CYP27B1* ^1215 T>C^). c.1215+2T>A was also created as a control by site-directed mutagenesis (CYP27B1^1215+2T>A^). CHO cells maintained in F12 with 10% FBS were transiently transfected with 20μg of the wild-type and mutant minigene constructs. Forty-eight hours after transfection, the cells were collected and RNA was extracted by quanidine thiocyanate-phenol-chloroform method and reverse-transcribed into cDNA for RT-PCR analysis using the same primer pairs and PCR conditions above.

## Results

### Clinical Characteristics

The diagnosis of 25-hydroxyvitamin D-1α-hydroxylase deficiency was made in each patient based on their clinical and biochemical features (Table 1). All of the patients required continued calcitriol treatment, indicating complete loss of 25-hydroxyvitamin D-1α-hydroxylase activity. Patients II-3 and IV-3 presented with severe hypophosphatemia associated with normal serum calcium, which is consistent with stage II rickets (Table 1).

### Sequence analysis of the *CYP27B1* gene

**Fig 1:**
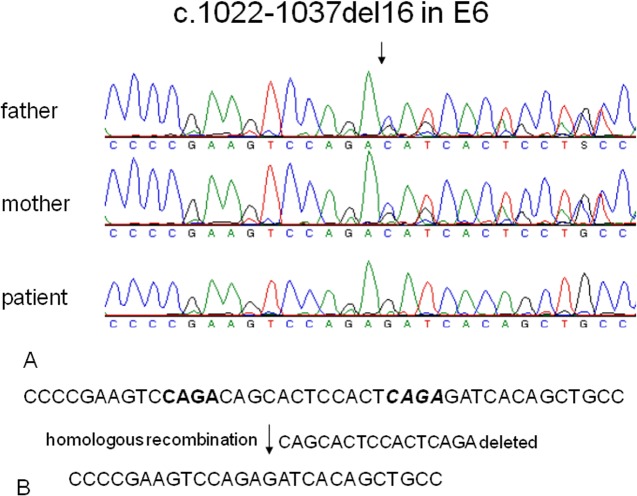
A novel deletion of 16-bp nucleotides in the human *CYP27B1* gene. (A) Sequence analysis shows a homozygous deletion of 16-bp nucleotides in exon 6 in a patient from family 1. Both of his parents carry a heterozygous deletion. (B) A schematic representation of the deletion. The deleted nucleotide sequence is underlined and the 4-bp nucleotide repeats flanking the deleted sequence are highlighted in bold.

**Fig 2:**
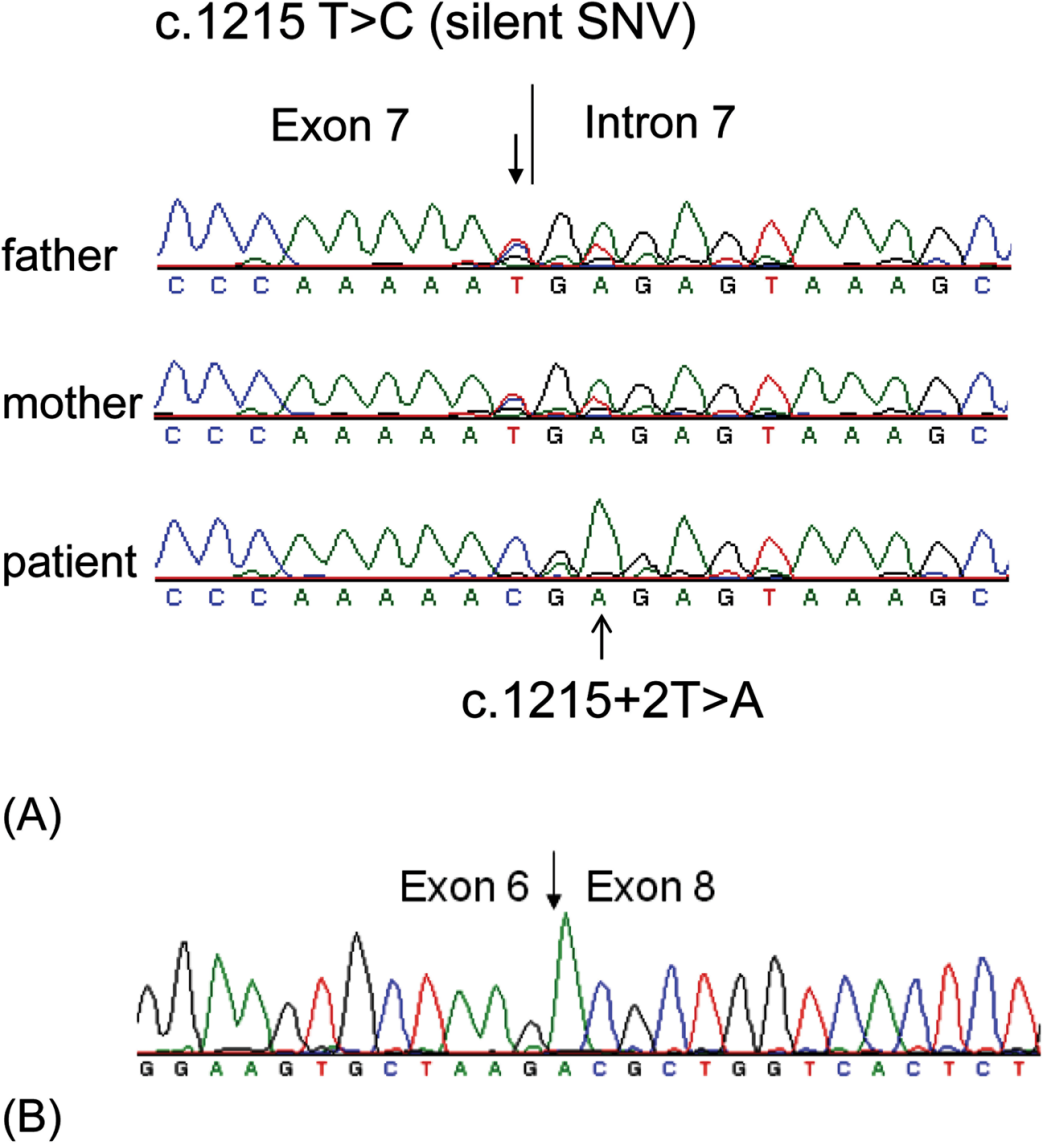
Novel splice site mutations in the human *CYP27B1* gene. (A) Sequence analysis of genomic DNA from peripheral lymphocytes. A homozygous mutation at the splice donor site of intron 7 (c.1215+2T>A) were found in a patient from family 3. A homozygous silent SNV (c.1215 T>C) at the end of exon 7 was also identified. His parents carry a heterozygous mutation at both these locations, demonstrating they are in cis and not in trans. The mutations are indicated by arrows. (B) Sequence analysis of cDNA from patient’s peripheral lymphocytes. The mutation at the c.1215+2T>A leads to skipping of exon 7, resulting in exons 6 and 8 joined together.

**Fig 3:**
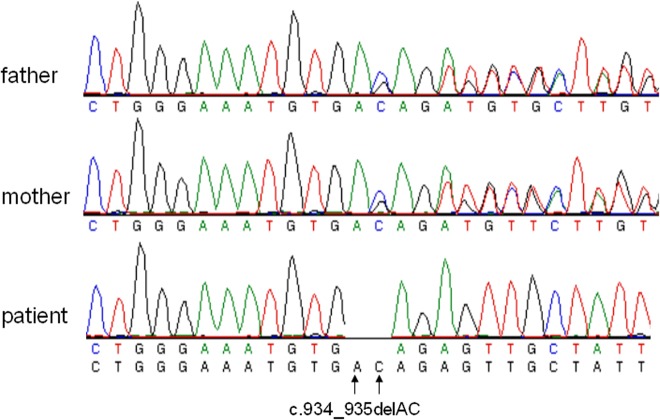
A novel deletion of 2-bp nucleotides in the human *CYP27B1* gene. A homozygous deletion of AC nucleotides (c.934_935delAC) in exon 5 was found in a patient from family 7. A heterozygous deletion was found in both of his parents. The deletion results in a frameshift and creates a premature TGA stop codon 19 amino acids downstream from the frameshift (p.T312RfsX19).

To identify the underlying genetic defects leading to 25-hydroxyvitamin D-1α-hydroxylase deficiency, we sequenced the entire coding region and intron-exon boundaries of the *CYP27B1* gene in the patients and their parents. Homozygous mutations in the *CYP27B1* gene were found in all the patients and heterozygous mutations were present in their asymptomatic parents (Table 1). Three novel mutations were identified: (a) A 16-bp deletion in exon 6 (c.1022-1037del16) in families I and II (Fig 1A). The intra-exon deletion of c.1022-1037del16 is likely caused by a homologous recombination due to CAGA repeats flanking the deleted sequence (Fig 1B). The deletion resulted in a frameshift and the creation of a premature stop codon 5 amino acids downstream from the frameshift (p.T341Rfs*5), leading to the functional inactivation of the *CYP27B1* gene; (b) c.1215 T>C (p.R379R) in the last nucleotide of exon 7 (Fig 2A) and a splice donor site mutation (c.1215+2T>A) in intron 7 (Fig 2A) in Families III and VI. Both mutations were found together in homozygous form in the patinets from the two unrelated families. They also segregated together in heterozygous form in the parental carriers; therefore they are likely to be in cis. Since c.1215 T>C does not change amino acid and is not present in both dbSNP and the University of Washington’s SNP database, it is likely a novel silent SNV; but it is not clear if it has any detrimental effect on pre-mRNA splicing. The splice site mutations caused exon 7 skipping in the mRNA from the patients’ leukocytes (Fig 2B). The skipping of exon 7 resulted in a shift of the downstream reading frame and a premature stop codon 57 amino acids from L380 (p.L380Afs*57). The transcripts may not be translated to the truncated protein due to nonsense-mediated decay. (c) A 2-bp deletion in exon 5 (c.934_935delAC) (Fig 3) in Family VII. This intra-exon deletion also resulted in a frameshift mutation, which created a premature stop codon 19 amino acids downstream from the frameshift (p.T312Rfs*19), leading to the functional inactivation of the *CYP27B1* gene. The previously reported c.195+2T>G mutation was found in the remaining three patients from families IV and V (Table 1). Since these mutations occur quite frequently in the Turkish patients, we did literature search of all the mutations found in the Turkish population and compared them with other ethnic groups. Among 9 mutations found in 17 patients from 12 unrelated families, 7 are unique mutations currently found only in the Turkish patients (Table 2).

**Table 2:** *CYP27B1* mutations in Turkish population.

Mutation	Turkish population	Other ethnic populations	References
c.1022-1037del16	2 patients from 2 unrelated families	not reported yet	current study
c.1215+2T>A	2 patients from 2 unrelated families	not reported yet	current study
c.1215 T>C (silent)	2 patients from 2 unrelated families (presence together with c.1215+2T>A)	not reported yet	current study
c.934_935delAC	One patient	not reported yet	current study
c.195+2T>G	5 patients from 3 unrelated families	not reported yet	current study, [28]
c.1079 C>A (p.S360*)	One patient	not reported yet	[28]
c.1166G>A (p.R389H)	One patient	White-USA	[19]
1319-1325dup CCCACCC	4 patients from 2 unrelated families	Polish, Chinese, Black, White, Hispanic, Pilipino	[19] [28]
c.403 C>T(p.Q135X)	One patient	not reported yet	http://espe2014abstracts.eurospe.org/hrp/0082/hrp0082P2-D3-308.htm

### Minigene analysis of the splicing mutations

**Fig 4:**
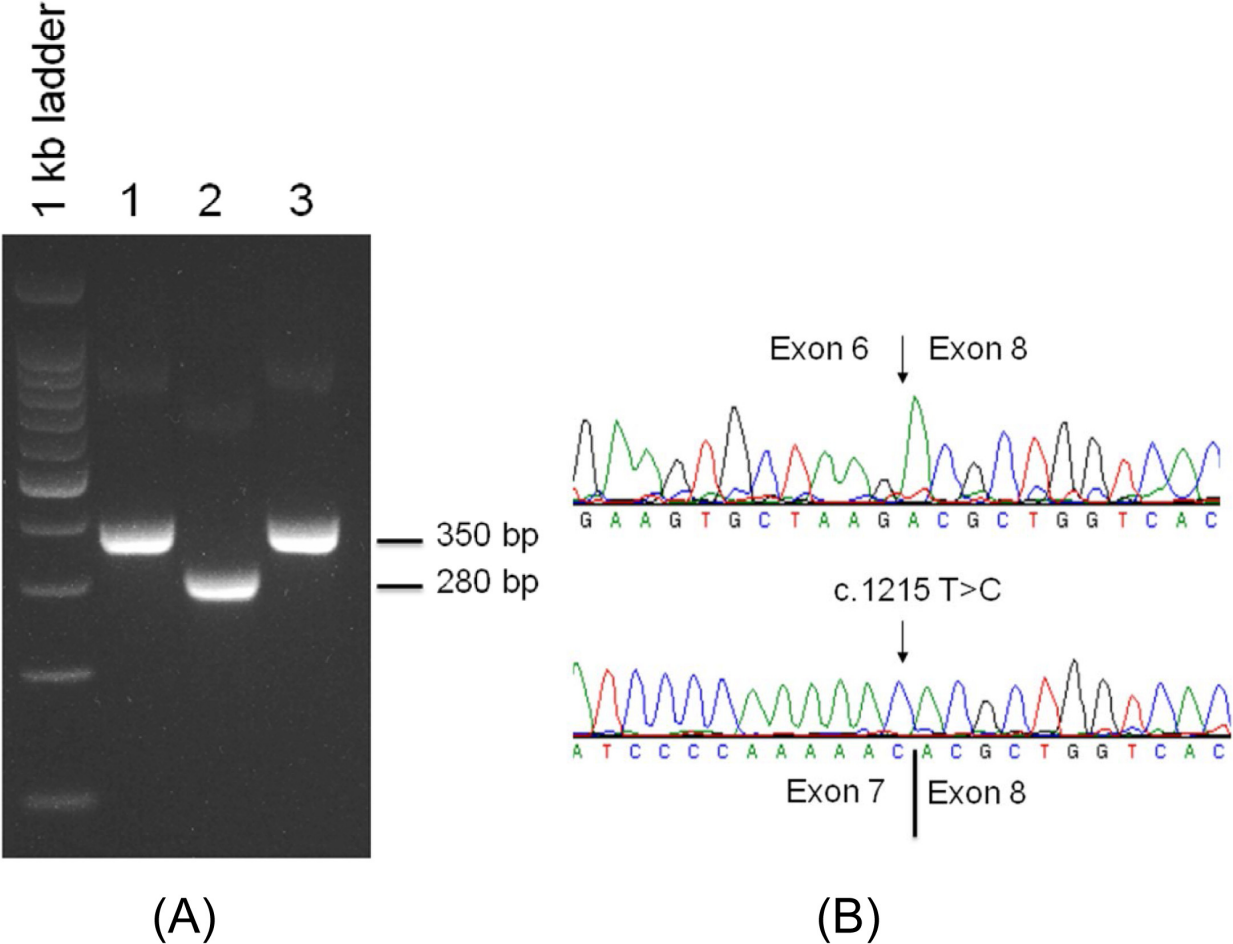
Minigene analysis of the splicing mutations. (A) *CYP27B1* ^WT^, CYP27B1^1215+2T>A^ and *CYP27B1* ^1215T>C^ constructs were transfected into CHO cells for *CYP27B1* minigene expression. RNA from transfected cells was reversed-transcribed to cDNA for RT-PCR analysis. Lane 1: *CYP27B1* ^WT^, lane 2: CYP27B1^1215+2T>A^, and lane 3: *CYP27B1* ^1215T>C^. (B) Sequencing analysis of cDNA fragments. Top panel: the 280 bp cDNA fragment from lane 2 lacks exon 7 (exon 7 skipping) due to 1215+2T>A mutation; Bottom panel: the 350 bp cDNA fragment contains 1215T>C silent mutation. This cDNA fragment is properly spliced containing exons 6, 7 and 8, indicating that the mutation has no effect on pre-mRNA splicing. Only exons 7 and 8 are shown together with 1215T>C silent mutation.

To investigate which splice site mutation (c.1215 T>C or c.1215+2T>A or both) caused aberrant pre-mRNA splicing, we constructed a wild-type *CYP27B1* mini-gene containing exons 6–8 and introns 6–7 (*CYP27B1* ^WT^), and two mutant mini-genes: *CYP27B1* ^1215T>C^ and CYP27B1^1215+2T>A^. The mini-gene constructs were transfected to CHO cells for mini-gene expression under CMV promoter. RNA was examined for splicing errors. As shown in Fig 4A, a 280-bp cDNA fragment was observed from CYP27B1^1215+2T>A^ construct whereas a 350- bp cDNA fragment was generated from both *CYP27B1* ^WT^ and *CYP27B1* ^1215T>C^ constructs. Sequencing analysis confirmed that the 280-bp cDNA fragment resulted from exon 7 skipping and the 350-bp cDNA fragment contained properly spliced exons 7 and 8 (Fig 4B). These data confirmed that the novel c.1215 T>C SNV has no effect on pre-mRNA splicing as opposed to the c.1215+2T>C mutation which led to the skipping of exon 7 and resultant frameshift and a premature stop codon. Therefore, the homozygous 1215+2 mutation is likely the pathogenic mutation for these patients, and the 1215T>C SNV is a benign/population SNP unique to these Turkish families.

## Discussion

In the present study, we have identified three novel mutations (c.1215+2T>A, c.1022-1037del16, and c.934_935delAC) and one previously reported mutation (c.195+2T>G). Each of c.1215+2T>A and c.1022-1037del16 mutations occurred twice in patients from different families. The c.195+2T>G was also first discovered in a Turkish patient [28] and, in the current study, the same mutation was found in 3 patients from two different families. Since these mutations occur quite frequently in the Turkish patients and have not been reported in other ethnic groups, they may unique in the Turkish population representing some kind of ‘founder mutation’ effect, given the high levels of consanguinity and the fact that most of the mutations are homozygous, as opposed to compound heterozygous in other populations. The clinical and genetic features of the patients are consistent with the lack of 25-hydroxyvitamin D-1α-hydroxylase activity and a classic pattern of autosomal recessive inheritance.

There are three stages of rickets based on biochemical and radiographic findings [29]. In the first stage, only serum calcium level is reduced with normal serum phosphorus and alkaline phosphatase. In the second stage, serum calcium level is normal but serum phosphorus level is low with high serum level of alkaline phosphatase. In the final stage of rickets, both serum calcium and phosphorus levels are low. Patients with severe hypophosphatemia with normal calcium and high PTH have been reported in two cases in our previous study [28]. In the current study, we reported two additional cases. The clinical presentations of these patients could lead to a wrong diagnosis of hypophosphatemic rickets. However, demonstration of low serum level of 1,25(OH)2 D and high PTH would confirm the diagnosis. The hypophosphatemia in VDDR-1A is a result of elevated PTH and renal excretion of phosphate, and indicates that the stage of rickets is at least 2. PTH reduces phosphate and increases calcium reabsorption from the proximal tubule of kidney, causing more phosphate and less calcium being excreted through the urine [30]. PTH also increases serum calcium by mobilizing calcium from bones through osteoclast-mediated bone resorption [30]. This is reflected by the very high serum ALP found in these patients. *Cyp27b1* knockout mice grow normally when they are maintained on a balanced diet containing 1,25(OH)_2_D. However, these mice rapidly develop rickets when a low phosphorus diet is given without additional 1,25(OH)_2_D despite maintaining normal serum calcium concentrations, a clinical picture that resembles stage II rickets in our cases [31].

A substantial proportion of gene mutations leading to human genetic disease are splice site mutations. More than 10% of disease-causing point mutations affect pre-mRNA splicing [32, 33]. Mutations at donor or acceptor splice sites can result in exon skipping, intron retention or insertions and deletions due to utilization of cryptic splice sites. There are 6 splice site mutations described in the *CYP27B1* gene: c.195+2T>G in intron 1[28], c.386+1G>A in intron 2 [23], c.589+1G>A in intron 3 [22], c.1136+1G>T in intron 6 [34], c.1215+1G>A in intron 7 [24], and c.1215+2T>A in intron 7 (current study). It has been confirmed that c.195+2T>G, c.386+1G>A and c.589+1G>A mutations lead to intron retention. The consequences of c.1136+1G>T and c.1215+1G>A have not, as of yet, been described. Since the c.1215+2T>A causes exon skipping in the current study and both c.1215+2T>A and c.1215+1G>A are located in the same splice donor site in intron 7, it is likely that c.1215+1G>A will result in exon skipping. Exon skipping is the most common consequence when the immediate vicinity of the affected exon-intron junctions is devoid of alternative splice-sites. Indeed, no cryptic splice donor sites were found in the exon 7 or intron 6. Splice site mutations or intra-exon deletions usually lead to a shift in the reading frame and creation of a premature stop codon, resulting in truncated proteins devoid of function. However, the functional loss of mutated allele can also be through nonsense-mediated decay [35]. In this case, when a premature stop codon is present, the entire mRNA is degraded. In either case, the functional activity of 25-hydroxyvitamin D-1α-hydroxylase is lost.

Generally, a good response to treatment with alfacalcidol or calcitriol (10–400 ng/kg/day) is expected in cases with VDDR-IA [14, 24]. However, withdrawal of treatment for ≥ 1 week would result in decrease in calcium and phosphorus and increase in ALP and PTH levels [26, 28]. In the present study, two siblings with c.195+2T>G mutation (Patients IV-3 and IV-4), who were not treated and followed up appropriately, had significant growth retardation. The girl with c.1215+2T>A mutation (Patient VI-3) was remarkably short as well. Consistently elevated PTH levels necessitated parathyroid scintigraphy to rule out secondary hyperparathyroidism, which turned out to be normal. On the other hand, the other patient with the same mutation (Patient III-3) demonstrated significant height gain after treatment. The lack of improvement in growth in some of our cases is likely due to poor compliance to treatment.

In conclusion, our findings demonstrate that (i) the spectrum of mutations in *CYP27B1* is expanding, (ii) the c.195+2T>G, c.1215+2T>A, and c.1022-1037del16 mutations might be unique to Turkish patients, (iii) the c.1215 T>C mutation is not detrimental to pre-mRNA splicing and may be considered as a novel SNP, and (iv) some patients with 1α-hydroxylase deficiency may not exhibit catch-up growth when compliance to treatment is not good.
